# Targeting the tumor-promoting microenvironment in MET-amplified NSCLC cells with a novel inhibitor of pro-HGF activation

**DOI:** 10.18632/oncotarget.18260

**Published:** 2017-05-29

**Authors:** Benjamin Y. Owusu, Shantasia Thomas, Phanindra Venukadasula, Zhenfu Han, James W. Janetka, Robert A. Galemmo, Lidija Klampfer

**Affiliations:** ^1^ Department of Oncology Southern Research, Birmingham, AL, 35205 USA; ^2^ Department of Biochemistry and Molecular Biophysics, Washington University School of Medicine, St. Louis, MO, 63110 USA

**Keywords:** HGF, MET, NSCLC

## Abstract

Targeted therapeutic agents, such as inhibitors of epithelial growth factor receptor (EGFR), have transformed the management of non-small cell lung cancer (NSCLC) patients. MET-amplified NSCLC cells display resistance to EGFR-targeting agents, but are addicted to MET signaling for survival and proliferation and are sensitive to MET inhibition. However, responsive cancer cells invariably develop resistance to MET-targeted treatment.

The tumor microenvironment plays a major role in resistance to anticancer therapy. We demonstrated that fibroblasts block the response of MET-amplified NSCLC cells to the MET kinase inhibitor, JNJ38877605 in an HGF-dependent manner. Thus, MET-amplified NSCLC cells become addicted to HGF upon pharmacological inhibition of MET. HGF restored phosphorylation of MET, EGFR and RON, and maintained pro-survival AKT and ERK signaling in MET-inhibited cells.

We developed a small molecule inhibitor of pro-HGF activation, SRI31215, which acts as a triplex inhibitor of the pro-HGF activating proteases matriptase, hepsin and HGF activator (HGFA). SRI31215 blocked crosstalk between tumor cells and fibroblasts and overcame fibroblast-mediated resistance to MET inhibition by preventing fibroblast-mediated reactivation of AKT and ERK signaling. Structurally unrelated triplex inhibitors of matriptase, hepsin and HGFA that we developed in parallel showed similar biological activity.

Our data suggest that simultaneous inhibition of HGF and MET is required to overcome resistance to MET inhibitors in MET-amplified NSCLC cells. This provides a rationale for the development of novel combination therapeutic strategies for the treatment of NSCLC patients with MET amplification.

## INTRODUCTION

Lung cancer remains a leading cause of cancer-related deaths worldwide. Oncogenic EGFR activation, commonly observed in non-small cell lung cancer (NSCLC), drives cell proliferation and survival. Indeed, EGFR tyrosine kinase inhibitors (TKIs) such as gefitinib and erlotinib have become an important therapeutic modality for the treatment of NSCLC patients with EGFR mutations [1–3]. However, innate or acquired resistance limits the efficacy of kinase inhibitors. The presence of KRas, BRaf and ERBB2 mutations predicts primary resistance to EGFR inhibitors [4, 5]. Acquired resistance to EGFR inhibitors has been associated most frequently with selection for secondary EGFR mutations, such as T790M in exon 20 [6, 7] and with MET amplification [8]. Second and third generation EGFR inhibitors have been developed and novel combination of these inhibitors with other therapies have been tested to overcome therapeutic resistance [9]. Additionally, drugs that target MET are in clinical trials to overcome MET-amplification-mediated resistance to EGFR inhibitors [10].

MET amplifications have been found in ∼20% of NSCLC patients with acquired resistance to gefitinib or erlotinib [8, 11]. MET-amplified lung cancer cells do not respond to EGFR inhibitors [12], but are highly sensitive to MET inhibition [13, 14]. Several MET kinase inhibitors have been evaluated in clinical trials [15], however acquired resistance remains a serious limitation of these drugs. Although the mechanisms of resistance to MET targeting drugs are not well understood, it has been shown that a switch to EGFR dependency can underlie the resistance to MET kinase inhibitors. Thus, one approach to optimize the anticancer activity and to prevent the growth of resistant clones in MET-amplified lung cancer cells is the combined therapy with MET and EGFR inhibitors [16, 17]. However, lung cancer cells with amplified MET and overexpressed EGFR frequently show limited response to combined treatment [16, 18], underscoring the necessity to identify additional mechanism of resistance.

In addition to cell-autonomous mechanisms of resistance, factors in the tumor microenvironment have been shown to block the response to therapy [19, 20]. Stroma-induced resistance to targeted therapy is frequently mediated by hepatocyte growth factor (HGF)-induced activation of MET [19–21]. HGF activates pro-survival signaling and promotes epithelial-mesenchymal transition of cancer cells, hindering the response to therapy. HGF has been shown to block the response of lung cancer cells to EGFR targeting drugs [22, 23] and increased levels of serum HGF predict poor progression-free survival and overall survival in NSCLC patients treated with EGFR inhibitors [24, 25]. Surprisingly, it has recently been shown that MET-amplified lung cancer cells become dependent on HGF for survival upon pharmacologic MET inhibition [26]. This indicates that both HGF and MET need to be targeted in MET-amplified NSCLC to prevent or to delay the onset of resistance.

Neutralizing anti-HGF antibodies that are being tested, alone or in combination with other therapies, appear to be well tolerated in clinical trials [27, 28]. They have shown encouraging results, yielding partial responses in cancer patients. However, there are currently no small molecule HGF inhibitors available for clinical trials. We developed and characterized two distinct series of low molecular weight inhibitors of HGF, which act as inhibitors of pro-HGF activation [29, 30].

HGF is secreted as a single chain inactive precursor called pro-HGF. Proteolytic conversion of pro-HGF to the heterodimeric active HGF is required to trigger ligand-dependent MET signaling. This is achieved by one of the trypsin-like serine proteases, matriptase, hepsin or HGF activator (HGFA), which are expressed by tumor cells [31]. A proteolytically inert mutant form of pro-HGF (uncleavable-HGF) or a truncated form of HGF that lacks the β-chain (NK4) displayed a competitive antagonism with HGF for MET binding. Both mutants suppressed proliferation, migration, and invasion of cancer cells and inhibited tumor growth and metastasis *in vivo* [32–35]. We demonstrated that triplex inhibitors of matriptase, hepsin and HGFA prevent activation of pro-HGF and inhibit HGF/MET signaling. SRI31215, a novel inhibitor of pro-HGF activation, blocked signaling between tumor-promoting fibroblasts and colon cancer cells and overcame fibroblast-mediated resistance to EGFR inhibitors in colon cancer cells [30, 36].

In this study, we established that recombinant HGF or HGF-producing fibroblasts protect MET-amplified NSCLC cells from MET-targeting therapy by maintaining AKT and ERK signaling in MET-inhibited cells. We demonstrated that novel inhibitors of HGF activation overcome fibroblast-mediated resistance to MET inhibition in MET-amplified lung cancer cells. This highlights the need to include agents blocking the biological activity of HGF, such as inhibitors of pro-HGF activation, into therapeutic regimens for MET-amplified NSCLC patients.

## RESULTS

### HGF rescues MET-amplified lung cancer cells from MET tyrosine kinase inhibition

**Figure 1:**
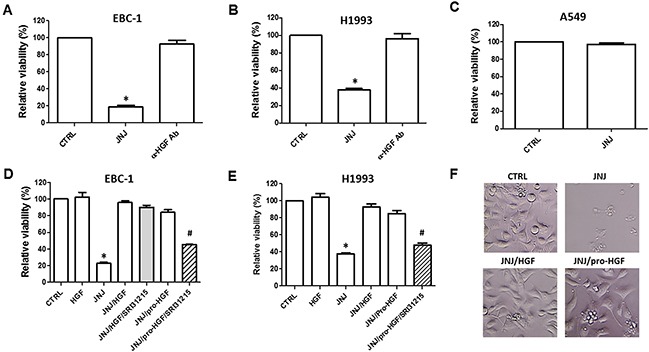
HGF rescues MET-amplified lung cancer cells from MET inhibition EBC-1 **(A)** and H1993 **(B)** MET-amplified lung cancer cells, and A549 cells **(C)** which do not have amplified MET were treated with JNJ38877605 (JNJ) (25 nM) or HGF neutralizing antibody (α-HGF Ab). Cell viability was determined after 72 h by CellTiter Glo^®^. **(D** and **E)** EBC-1 and H1993 cells were treated with JNJ38877605 (25 nM), HGF (100 nM) or pro-HGF (100 nM) as indicated. Cell viability was determined by CellTiter Glo^®^. **(F)** Representative images of EBC-1 cells treated with JNJ38877605 alone or with HGF or pro-HGF after 72 h of treatment.

Whereas MET-amplified lung cancer cells do not respond to EGFR inhibition, their growth is strongly inhibited by MET tyrosine kinase inhibitors, confirming that these cells are addicted to MET signaling [13, 14]. Accordingly, the selective MET tyrosine kinase inhibitor, JNJ38877605, inhibits the viability of two MET-amplified NSCLC cell lines, EBC-1 and H1993 (Figure 1A and 1B). Inhibition of HGF using an HGF-specific neutralizing antibody had no impact on the viability of EBC-1 or H1993 cells (Figure 1A and 1B), confirming that MET amplification triggers ligand-independent MET signaling. We showed that JNJ38877605 inhibits the viability of EBC-1 cells in a dose- and time-dependent manner (Supplementary Figure 1A and 1B). In contrast, A549 cells, which do not harbor amplified MET, did not respond to the growth-inhibitory effect of JNJ38877605 (Figure 1C). This demonstrates that MET-amplified lung cancer cells display selective sensitivity to MET kinase inhibition.

HGF has been shown to mediate resistance to several targeted therapeutic agents such as inhibitors of EGFR, BRaf and HER2 [20, 37]. Here we show that HGF also blocks the response to JNJ38877605, a specific MET kinase inhibitor, in MET-amplified EBC-1 and H1993 cells (Figure 1D-1F). HGF increased the survival of MET-inhibited cells in a dose-dependent manner (Supplementary Figure 1C). Pro-HGF, the inactive precursor of HGF, was as potent as HGF in protecting EBC-1 and H1993 cells from JNJ38877605-induced cell death (Figure 1D-1F), confirming that these cell lines express enzyme(s) that can activate pro-HGF into its mature form.

**Figure 2:**
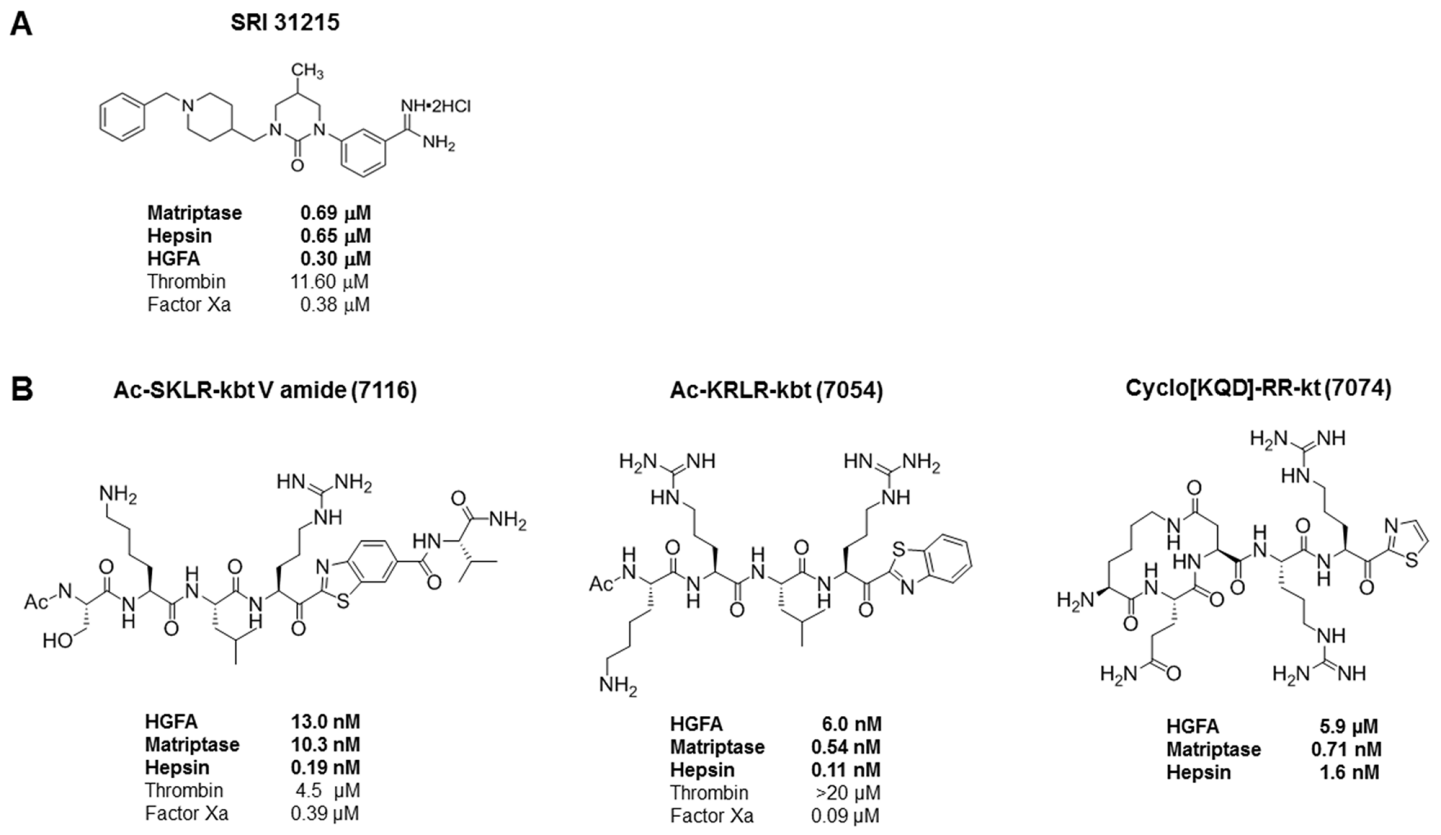
Molecular structure and activity of SRI31215 (A) and peptide inhibitors of pro-HGF activation, ZFH7054, ZFH7074 and ZFH7116 (B) The IC_50_ values for matriptase, hepsin and HGFA are indicated in bold.

These data indicated that MET-amplified NSCLC cells rely on HGF for survival upon pharmacological inhibition of MET. We have shown earlier that SRI31215, a triplex inhibitor of matriptase, hepsin and HGFA, (Figure 2), inhibits pro-HGF activation [36]. Here we show that SRI31215 blocks the pro-survival activity of pro-HGF in EBC-1 and H1993 cells (Figure 1D and 1E). Consistent with its mode of action, SRI31215 does not impact the pro-survival activity of mature HGF (Figure 1D) [36].

The levels of matriptase and hepsin, two of the pro-HGF converting enzymes, are significantly elevated in lung cancer compared to normal lung tissue (Supplementary Figure 2). Moreover, the levels of HGF are increased in lung cancer tissue compared to normal lung mucosa (Supplementary Figure 2), suggesting that inhibition of pro-HGF activation is a valid approach to inhibit HGF/MET signaling.

### Inhibition of pro-HGF activation overcomes fibroblast-mediated resistance to MET kinase inhibition

HGF is a key growth factor in the tumor micro-environment that inhibits the response of cancer cells to targeted therapy [20]. Cancer-associated fibroblasts are the main source of pro-HGF in the tumor microenvironment, therefore we tested whether inhibitors of pro-HGF activation can overcome fibroblast–mediated resistance to MET-targeted therapy. These experiments were performed in the absence of serum, which contains enzymes that can cleave and activate pro-HGF.

**Figure 3:**
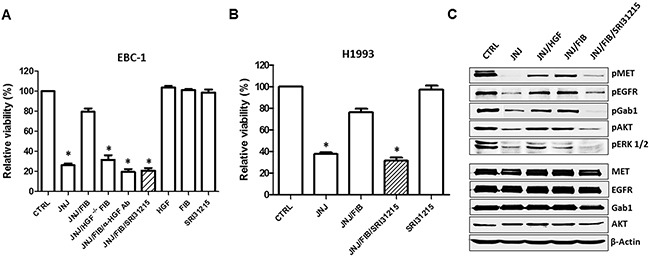
Inhibition of pro-HGF activation overcomes fibroblast-mediated resistance to MET tyrosine kinase inhibition (**A)** EBC-1 cells were treated with JNJ38877605 (25 nM) alone or in the presence of conditioned medium (CM) from WI38 fibroblasts (FIB). CM was also prepared from fibroblasts with silenced HGF (HGF^−/−^ FIB), or from fibroblasts cultured with HGF neutralizing antibody (α-HGF Ab) or SRI31215 (10 μM) as indicated. Cell viability was determined by CellTiter Glo^®^72 h after treatment. **(B)** H1993 cells were treated with JNJ38877605 (25 nM), fibroblast CM (FIB) and SRI31215 (10 μM) as indicated and cell viability was determined after 72 h. **(C)** Serum-starved EBC-1 cells were treated with JNJ38877605, recombinant HGF (100 nM), FIB and SRI31215 (10 μM) as indicated for 6 hours. Cell lysates were analyzed by immunoblotting for phospho- and total MET, EGFR, Gab1, AKT and ERK 1/2. *, *p* < 0.05 compared to JNJ/FIB.

Consistent with our finding that HGF and pro-HGF block the activity of JNJ38877605 (Figure 1D and 1E), conditioned medium from WI38 lung fibroblasts which produce pro-HGF (Supplementary Figure 3A) rescued EBC-1 and H1993 cells from JNJ38877605-induced cell death (Figure 3A and 3B). We showed that fibroblasts with silenced HGF (Supplementary Figure 3A) failed to inhibit the response of EBC-1 cells to JNJ38877605 (Figure 3A). Accordingly, HGF neutralizing antibodies blocked the ability of fibroblasts to protect EBC-1 cells from JNJ38877605-induced cell death (Figure 3A). These experiments established that fibroblasts inhibit JNJ38877605-induced cell death through HGF. Consistent with published data [17], HGF or fibroblasts did not further stimulate MET signaling (Supplementary Figure 3B) or promote the growth of MET-amplified EBC-1 (Figure 3A).

Like HGF neutralizing antibodies, SRI31215 blocked the ability of fibroblasts to rescue EBC-1 and H1993 cells from JNJ38877605-induced cell death (Figure 3A and 3B). Accordingly, the substrate-based peptide inhibitors of matriptase, hepsin and HGFA, ZFH7054, ZFH7074 and ZFH7116 (Figure 2) restored the sensitivity of EBC-1 cells to JNJ38877605 in the presence of fibroblasts and overcame the ability of pro-HGF to rescue EBC-1 cells from MET inhibition (Supplementary Figure 3C). Treatment of EBC-1 cells with SRI31215, ZFH7054, ZFH7074 or ZFH7116 alone did not have any impact on the viability of EBC-1 cells (Supplementary Figure 3D), confirming that these compounds do not target cancer cells directly, but inhibit the crosstalk between cancer cells and fibroblasts.

To understand the mechanism whereby HGF elicits resistance to JNJ38877605, EBC-1 cells were treated with JNJ38877605 alone or in the presence of recombinant HGF or fibroblasts and SRI31215. As expected, JNJ38877605 inhibited the expression of p-MET, p-EGFR, p-Gab1, p-ERK 1/2 and p-AKT (Figure 3C). We demonstrated that HGF or pro-HGF-producing fibroblasts restored phosphorylation of MET, EGFR and Gab1 in JNJ38877605-treated EBC-1 cells. Accordingly, HGF or fibroblasts reactivated ERK1/2 and AKT signaling upon pharmacological MET inhibition by JNJ38877605. Importantly, SRI31215 blocked fibroblast-induced MET and EGFR signaling and AKT and ERK activation in MET-inhibited cells (Figure 3C), consistent with its ability to sensitize EBC-1 and H1993 cells to JNJ38877605 (Figure 3A and 3B). The levels of total MET, EGFR, Gab1 and AKT were not altered by the treatments (Figure 3C). Treatment of cells with HGF, fibroblasts or SRI31215 alone did not alter the expression or the activity of MET, EGFR, Gab1 or AKT (Supplementary Figure 3B).

Collectively, our data show that fibroblasts elicit resistance to MET-targeted therapy through HGF in MET-amplified lung cancer cells. We showed that HGF is sufficient to sustain MET and EGFR signaling and downstream activation of ERK and AKT upon MET inhibition. Inhibitors of pro-HGF that we developed blocked the pro-survival activity of fibroblasts, indicating that dual inhibition of MET and HGF is required to overcome resistance to MET-targeted therapy in MET-amplified NSCLC.

### HGF supports the migration of MET-amplified EBC-1 lung cancer cells upon MET tyrosine kinase inhibition

**Figure 4:**
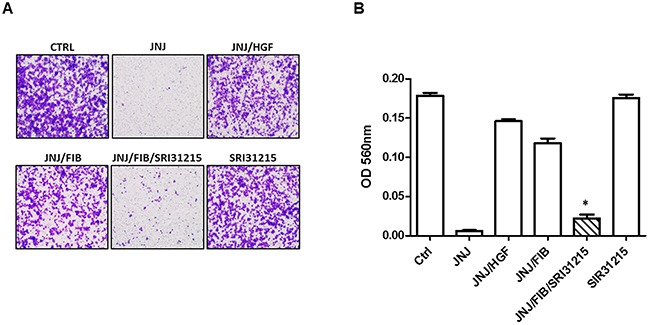
HGF upholds the migration of MET-addicted EBC-1 lung cancer cells upon MET tyrosine kinase inhibition Migration of EBC-1 cells was assessed by a transwell migration assay. EBC-1 cells were treated with JNJ38877605 (25 nM) in the absence or presence of recombinant HGF or conditioned medium from fibroblasts (FIB) and SRI31215 (10 μM) for 24 h. **(A)** Representative images of stained cells that migrated across the polycarbonate membrane. **(B)** Relative migration of cells was determined by the optical density (OD) of stained cells at 560 nm.*, *p* < 0.05 compared to JNJ/FIB.

MET activation promotes growth, survival and stimulates epithelial-mesenchymal transition which leads to enhanced migration of cancer cells [38]. Indeed, the migration of MET-amplified EBC-1 cells was completely inhibited by the MET tyrosine kinase inhibitor, JNJ38877605 (Figure 4). The inhibitory effect of JNJ38877605 on the migration of EBC-1 cells was prevented by either recombinant HGF or by WI38 lung fibroblasts (Figure 4). This result established that MET-amplified NSCLC cells become dependent on HGF not only for survival, but also for migration when MET is inhibited.

We have shown previously that SRI31215 impedes crosstalk between colon cancer cells and fibroblasts and thus inhibits the tumor-promoting activities of fibroblasts in colon cancer [36]. Here we demonstrate that SRI31215 blocks the ability of fibroblasts to restore the migration of MET-inhibited EBC-1 cells (Figure 4). The peptide inhibitors of pro-HGF activation, ZFH7054, ZFH7074 and ZFH7116 (Figure 2) which target the three pro-HGF activating enzymes [29], also blocked fibroblast-mediated migration of MET-inhibited EBC-1 cells (Supplementary Figure 4). Treatment of cancer cells with inhibitors of pro-HGF activation alone had no impact on the migration of EBC-1 cells (Figure 4 and Supplementary Figure 4).

Thus, while HGF (or fibroblasts) do not further stimulate MET signaling (Supplementary Figures 3B and 5A) or promote the viability (Figure 3) in MET-amplified cancer cells, our results established that MET-amplified NSCLC cells become addicted to HGF upon pharmacological MET inhibition. HGF-producing fibroblasts promote the survival and migration of MET-amplified lung cancer cells when MET is inhibited. We demonstrated that novel inhibitors of pro-HGF processing block crosstalk between tumor cells and fibroblasts and thus restrain the tumor-promoting activity of fibroblasts.

### HGF-induced signaling in MET-inhibited cells

To understand how HGF signals in MET-amplified lung cancer cells when MET kinase activity is inhibited, we used the Proteome Profiler Human Phospho-RTK array (R&D Systems) to investigate the phosphorylation profiles of 49 different receptor tyrosine kinases. Additionally, we used Phospho-Kinase Array (R&D Systems) containing 46 kinases and their substrates.

**Figure 5:**
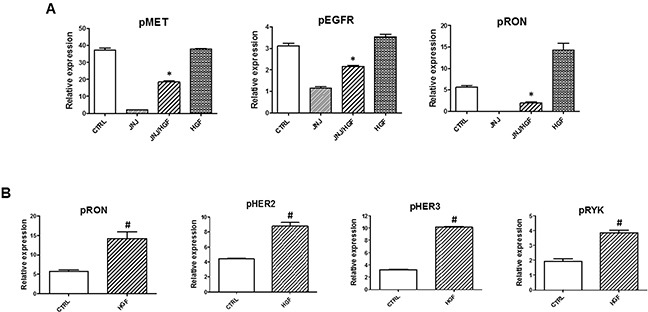
HGF-induced signaling in MET inhibited NSCLC cells EBC-1 cells were treated with JNJ38877605 (25 nM) or HGF (100 nM) alone, or with a combination of JNJ38877605 and HGF for 6 hours. Cell lysates were prepared and subjected to antibody-based human phospho-receptor tyrosine kinase (RTK) array as described in methods to analyze the phosphorylation profiles of receptor tyrosine kinases. *, *p* < 0.05 compared to JNJ38877605 (JNJ); ^#^, *p* < 0.05 compared to CTRL.

Inhibition of MET kinase by JNJ38877605 blocked MET phosphorylation, however we showed that JNJ38877605 also significantly reduced phosphorylation of EGFR, RON (Figure 5A), AXL and RET (Supplementary Figure 5A). This suggests that MET cross-phosphorylates these RTKs in MET-amplified EBC-1 cells. Indeed, MET interacts with several other receptor tyrosine kinases (RTKs) and MET heterodimers with EGFR, RON, HER2 and HER3 have been found abundant in MET-amplified cancer cells [39].

HGF restored phosphorylation of MET, EGFR and RON in MET-inhibited EBC-1 cells (Figure 5A, Supplementary Figure 5D), but did not rescue phosphorylation of AXL or RET (Supplementary Figure 5A) in JNJ38877605-treated cells. The array analysis confirmed that HGF restored activation of ERK and AKT in MET-inhibited cells (Supplementary Figure 5B). In addition, we found that WNK1, a prosurvival kinase that signals through ERK5 and Wnt [40], is highly activated in MET-amplified EBC-1 cells (Supplementary Figure 5C). Inhibition of MET kinase by JNJ38877605 significantly reduced phosphorylation of WNK1 in EBC-1 cells and HGF restored its activity in MET inhibited cells. In contrast, the levels of HSP60 were not restored by HGF in MET- inhibited cells (Supplementary Figure 5C). This indicates that novel mechanisms, such as WNK1 activation, may contribute to prosurvival activity of HGF in MET-amplified NSCLC cells and maintain the tumor-promoting crosstalk between tumor cells and cancer- associated fibroblasts. The potential role of WNK1 in HGF signaling and in HGF/fibroblast-dependent resistance to targeted therapy needs to be confirmed in other NSCLC cell lines.

How does HGF signal in MET-inhibited cells? It is possible that due to MET overexpression in MET-amplified cells there is always some residual MET in MET- inhibited cells, which responds to HGF stimulation. Indeed, we showed that HGF reactivates MET in JNJ38877605-treated cells (Figure 3 and Figure 5). However, we cannot exclude the possibility that HGF, at least in part, promotes survival of MET- amplified cells in a MET-independent manner. In fact, HGF stimulation increased phosphorylation of MET-related receptor RON, HER2, HER3 and RYK, which is likely to contribute to the pro-survival activity of HGF (Figure 5B).

## DISCUSSION

The tumor-microenvironment plays a major role in resistance to targeted therapy. In this study we demonstrated that HGF, commonly produced by cancer-associated fibroblasts, confers resistance to MET kinase inhibitors in MET-amplified NSCLC cells. We showed that a novel inhibitor of pro-HGF activation, SRI31215, blocks the crosstalk between cancer cells and fibroblasts and overcomes resistance to anti-MET therapy in MET-amplified NSCLC cells.

The availability of targeted therapeutic options, such as EGFR inhibitors, has improved the management of patients with EGFR mutant NSCLC. However, only a fraction of patients shows a good clinical response to EGFR inhibitors and virtually all patients develop resistance to therapy [41–43]. Acquired resistance to EGFR inhibitors has been associated most frequently with selection for secondary EGFR mutations, such as T790M in exon 20 [6, 7] and MET amplifications [8]. MET-amplified lung cancers, which display ligand-independent MET activation, are addicted to MET signaling and are extremely sensitive to MET inhibition [13]. Nevertheless, acquired resistance to MET kinase inhibitors remains a major limitation of these drugs. In some cases, a switch to EGFR dependency has been shown to underlie the resistance to MET kinase inhibitors [16, 17]. However, some lung cancer cells with amplified MET and overexpressed EGFR fail to respond to combined treatment with EGFR and MET inhibitors or develop resistance to dual EGFR/MET inhibition [44]. Indeed, resistance to dual EGFR/MET inhibition has recently been reported in a MET-amplified NSCLC patient [45].

The tumor microenvironment has been shown to elicit resistance to therapy. HGF, which is commonly overexpressed in the tumor microenvironment, is a frequent source of resistance to targeted therapy [19–21]. HGF activates MET, expressed on tumor cells, and drives resistance to EGFR-targeted therapy by triggering ERK and PI3K/AKT pro-survival signaling and by promoting epithelial-mesenchymal transition [20]. Elevated levels of circulating HGF are associated with a mesenchymal tumor type, poor patient prognosis and resistance to EGFR inhibitors in lung cancer patients [11, 25]. Notably, EGFR inhibitors have been shown to elevate plasma levels of HGF in NSCLC patients [46], which may be due to therapy-induced recruitment of fibroblasts. HGF is also induced in airway epithelium by smoking [47], by far the biggest cause of lung cancer.

Here we demonstrated that HGF also drives resistance to anti-MET therapy in MET-amplified lung cancer cells (Figure 1 and Figure 3). Thus, MET-amplified NSCLC cells become addicted to HGF upon MET inhibition. HGF reactivates MET, EGFR and RON phosphorylation and restores AKT, ERK and WNK1 signaling upon MET inhibition (Figure 3, Figure 5, Supplementary Figure 5), promoting the survival of MET-inhibited cancer cells. Thus, simultaneous inhibition of HGF and MET may be required to prevent resistance to targeted therapy in MET-amplified NSCLC cells.

Neutralizing anti-HGF antibodies that are being tested, alone or in combination with other therapies, appear to be well tolerated. They have shown encouraging results, yielding partial response in cancer patients [28, 48–51]. There are currently no known small molecule inhibitors of HGF available, which have several advantages compared to therapeutic antibodies [52]. We developed SRI31215, a novel small molecule inhibitor of pro-HGF activation, which is the rate-limiting step in HGF/MET signaling [30]. HGF is synthesized and secreted by tumors or, more commonly, by stromal cells as an inactive precursor, pro-HGF. SRI31215 is a triplex inhibitor of the three serine proteases, matriptase, hepsin and HGFA, each of them being sufficient for the processing of pro-HGF into the biologically active ligand [31]. Structurally distinct triplex inhibitors of matriptase, hepsin and HGFA that we developed in parallel (Figure 2) exert similar biological activity as SRI31215 (Supplementary Figures 3 and 4). SRI31215 and the peptide inhibitors mimic the activity of Hepatocyte Growth Factor Activator Inhibitors (HAI-1/2), which act as endogenous inhibitors of pro-HGF activation, and inhibit matriptase, hepsin and HGFA [31, 53]. While HGF-activating enzymes are upregulated in lung cancer (Supplementary Figure 2), the levels of HAI-1/2 are commonly reduced in cancer tissue, resulting in increased activation of HGF. Reduced expression of the HAIs is associated with advanced stage disease and poor prognosis in many cancers [54–60].

We demonstrated previously that SRI31215 effectively inhibits HGF-dependent MET activation, proliferation, epithelial-mesenchymal transition and migration of cancer cells and overcomes fibroblast-mediated resistance to EGFR inhibitors in colon cancer cells [36]. Here we established that SRI31215, and peptide-based inhibitors of pro-HGF activation, block the tumor-promoting activity of fibroblasts and overcome the resistance of MET-amplified lung cancer cells to anti-MET therapy (Figures 3 and 4). Our preliminary data indicate that SRI31215 also overcomes resistance to double MET/EGFR inhibition (data not shown).

Several MET kinase inhibitors have been developed and have entered clinical trials. In preclinical studies cancer cells rapidly develop resistance to MET kinase inhibitors, which will probably translate to resistance in patients. It has been demonstrated that discontinuation of MET tyrosine kinase inhibitors results in increased and prolonged MET activation, coupled to enhanced tumor growth *in vitro* and *in vivo* [61], suggesting that MET inhibitors will need to be combined with other therapies [62].

The mechanisms whereby cancer cells develop resistance to MET kinase inhibitors are not well understood. Recently, a lung adenocarcinoma patient with MET amplification developed resistance to dual anti-EGFR/MET therapy due to an acquired MET^D1228V^ mutation [45]. We demonstrated that HGF, commonly overexpressed in the tumor microenvironment, confers resistance to MET- targeted therapy in MET-amplified NSCLC cells. Thus, small molecule inhibitors of pro-HGF activation such as those described herein, offer a new approach to restore sensitivity to MET or MET/EGFR inhibition in MET-amplified NSCLC patients. Plasma levels of HGF can serve as a biomarker to select lung cancer patients that would benefit from drugs that counteract the biological activity of HGF.

In summary, our data underscore a key role of the tumor microenvironment in acquired resistance to MET kinase inhibitors in MET-amplified NSCLC cells. We demonstrated that MET-amplified NSCLC cells become addicted to HGF under pharmacological MET inhibition. Because HGF is frequently overexpressed in the tumor microenvironment, our work highlights the need to include inhibitors of biological activity of HGF into therapeutic regimen in MET-amplified NSCLC patients.

## MATERIALS AND METHODS

### Materials

Primary antibodies used were anti-MET, anti-pMET, anti-EGFR, anti-pEGFR, anti-GAB1, anti-pGAB1, anti-AKT, anti-pAKT, anti-pERK1/2, (Cell Signaling Technology) and anti-β-actin (Sigma). The antibody arrays used were Proteome Profiler Human Phospho-Receptor Tyrosine Kinase (RTK) (ARY001B) and Phospho-Kinase Array (ARY003B) kits from R&D Systems. Recombinant HGF and pro-HGF were purchased from R&D Systems. The MET kinase inhibitor, JNJ38877605 was purchased from Selleck Chemicals (Selleckchem). HGF-specific and nonspecific (NSP) siRNAs were purchased from Dharmacon.

### Cell culture

A549, WI38 and H1993 cells were obtained from ATCC and EBC-1 cells were purchased from XenoTech, KS. EBC-1, A549 and WI38 cells were maintained in minimum essential medium (MEM) and H1993 cells were cultured in RPMI medium. Growth media were supplemented with 10% fetal bovine serum, L-glutamine and antibiotics and cells were maintained under standard cell culture conditions at 37°C and 5% CO_2_. Conditioned medium from fibroblasts was prepared from 18Co fibroblasts as reported before [36]. Briefly, fibroblasts were maintained in complete medium until they reached confluence. The presence of HGF in conditioned media was confirmed by immunoblotting and by ELISA as described before [36]. Confluent cultures were rinsed and maintained in serum-free MEM medium for another 36 hours. Cell supernatants were collected, centrifuged and used immediately or were aliquoted and stored at −80°C.

### Transwell migration assay

Cytoselect cell migration assay kit was purchased from Cell Biolabs and the assay was performed according to the manufacturer's instructions. EBC-1 cells were seeded in transwells (8 μM pore size inserts) at a density of 7.5×10^4^ cells/well in serum-free media. Transwells were placed in wells (24-well format) containing 10% serum-containing media and incubated for 24h. Cells that migrated were stained for 10 minutes at room temperature, transferred to wells containing 200 μl of extraction buffer and incubated for 10 minutes on a shaker. The optical density (OD) of stained cells was measured at 560 nm.

### Viability assays

CellTiter Glo^®^ luminescent assay kit from Promega was used to assess cell viability. Cells were seeded in 96-well plates at a density of 1×10^4^ cells per well and treated as indicated. Cell viability was assessed 24, 48 and 72 hours after treatment, following the manufacturer's protocol.

### Immunoblotting

Immunoblotting was performed using standard procedures. Proteins were separated by SDS-PAGE. Membranes were blocked with 5% non-fat milk in Tris-buffered saline with Tween 20 (TBST) for 1 hour at room temperature (RT), and immunoblotted with primary antibodies in 5% BSA-TBST overnight at 4°C. Following washing with TBST buffer, membranes were incubated with horseradish peroxidase-conjugated secondary antibodies for 1 hour and then washed with TBST. Membranes were developed using the ECL chemiluminescent detection system (Amersham).

### Phosphokinase arrays

The phosphorylation profiles of kinases and their protein substrates were determined using Proteome Profiler Human Phospho-Receptor Tyrosine Kinase (RTK) and Human Phospho-Kinase Array kits (R&D Systems), following the manufacturers protocol. Serum-starved EBC-1 cells were treated with JNJ38877605 for 6h in the absence or presence of recombinant HGF. Cells were rinsed with PBS and then solubilized with lysis buffer at a concentration of 1×10^7^ cells/mL. Array membranes that had been spotted with capture antibodies were blocked with blocking buffer and then incubated with 300 μg of cell lysates overnight. Arrays were washed to remove unbound proteins followed by incubation with detection antibodies conjugated to horseradish peroxidase. Phosphorylated proteins were detected using chemiluminescent reagents and quantified using the Image Quant LAS 4000 array analysis software (GE Healthcare Life Sciences).

### HGF silencing

EBC-1 cells were seeded in a 6-well plate DMEM growth medium. Upon reaching 80-90% confluence, growth medium was changed to Opti-MEM and cells were transfected with HGF-specific siRNA (Dharmacon) using Lipofectamine 2000 (Invitrogen). Non-specific (NSP), nontargeting siRNA (Dharmacon) was used as a negative control. Both HGF and NSP siRNAs were delivered at a final concentration of 25 nM. Growth medium was changed to DMEM without antibiotics after overnight transfection. HGF silencing was confirmed by immunoblotting.

### Statistical analyses

All experiments were repeated at least three times. Results are expressed as the mean ± SEM as indicated. Statistical analyses using two-tailed student's t-test were performed with the GraphPad Prism 5.0 software. *p* < 0.05 was considered statistically significant.

## SUPPLEMENTARY FIGURES


