# Supplementary material for: Targeting the tumor-promoting microenvironment in MET-amplified NSCLC cells with a novel inhibitor of pro-HGF activation
Source: Oncotarget. 2017 May 29;8(38):63014–25. doi: 10.18632/oncotarget.18260 (PMC5609899; doi:10.18632/oncotarget.18260)
Supplement: Supplementary file 1 [file oncotarget-08-63014-s001.pdf]

Targeting the tumor-promoting microenvironment in MET-amplified NSCLC cells with a novel inhibitor of pro-HGF activation

SUPPLEMENTARY MATERIALS

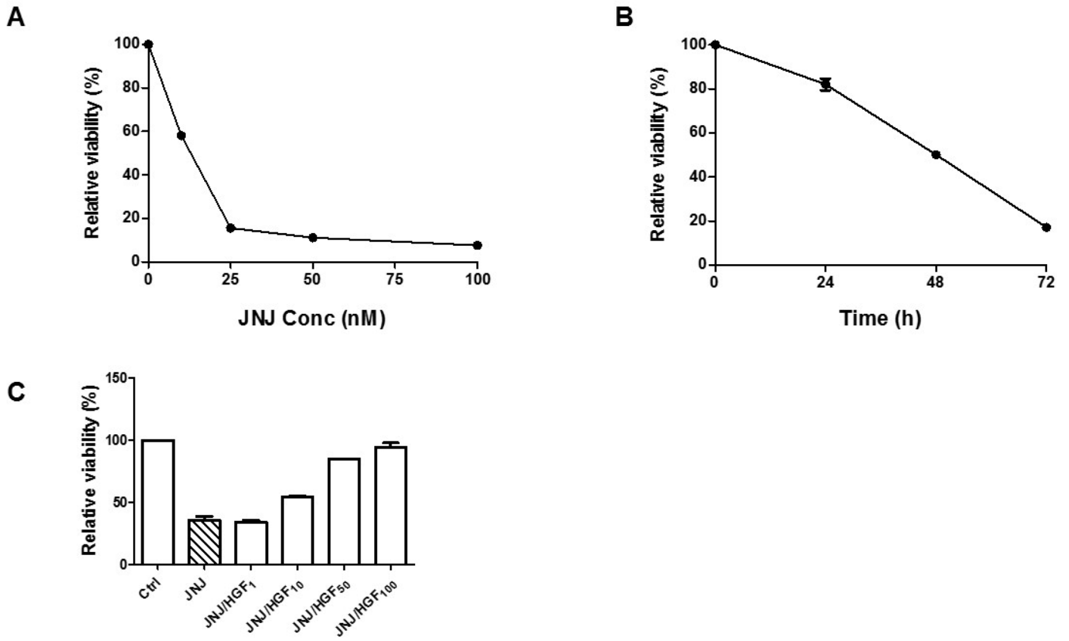

Supplementary Figure 1: JNJ38877605 inhibits viability of EBC-1 cells in a concentration- and a time-dependent manner. (A) EBC-1 cells were treated with increasing concentrations of JNJ38877605 (0, 10, 25, 50 and 100 nM) for 72 h. **(B)** EBC-1 cells were treated with 25 nM JNJ38877605 for 24, 48 and 72 h. Cell viability was determined by CellTiter Glo® assay. **(C)** HGF rescues MET inhibited cells in a concentration-dependent manner (1-100 nM).

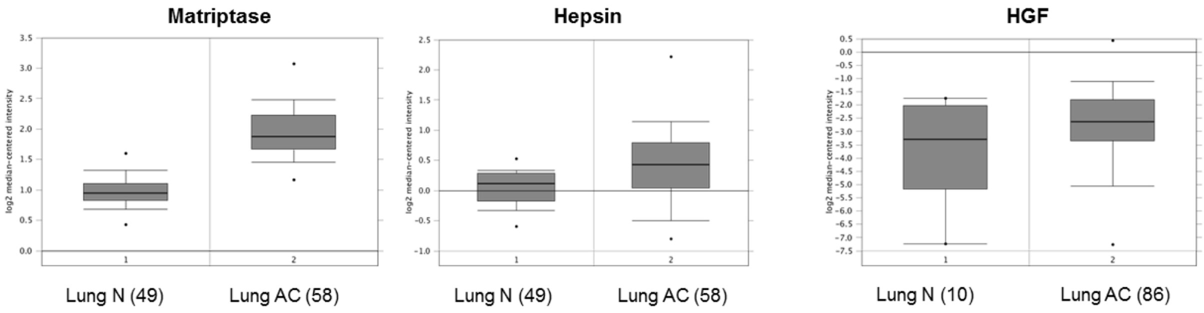

Supplementary Figure 2: Differential expression of matriptase and hepsin [65] and HGF [66] in normal (N) and lung adenocarcinoma (AC) tissues as surveyed by Oncomine analysis. *, $p < 0.05$ compared to CTRL or JNJ/HGF; #, $p < 0.05$ compared to JNJ/pro-HGF.

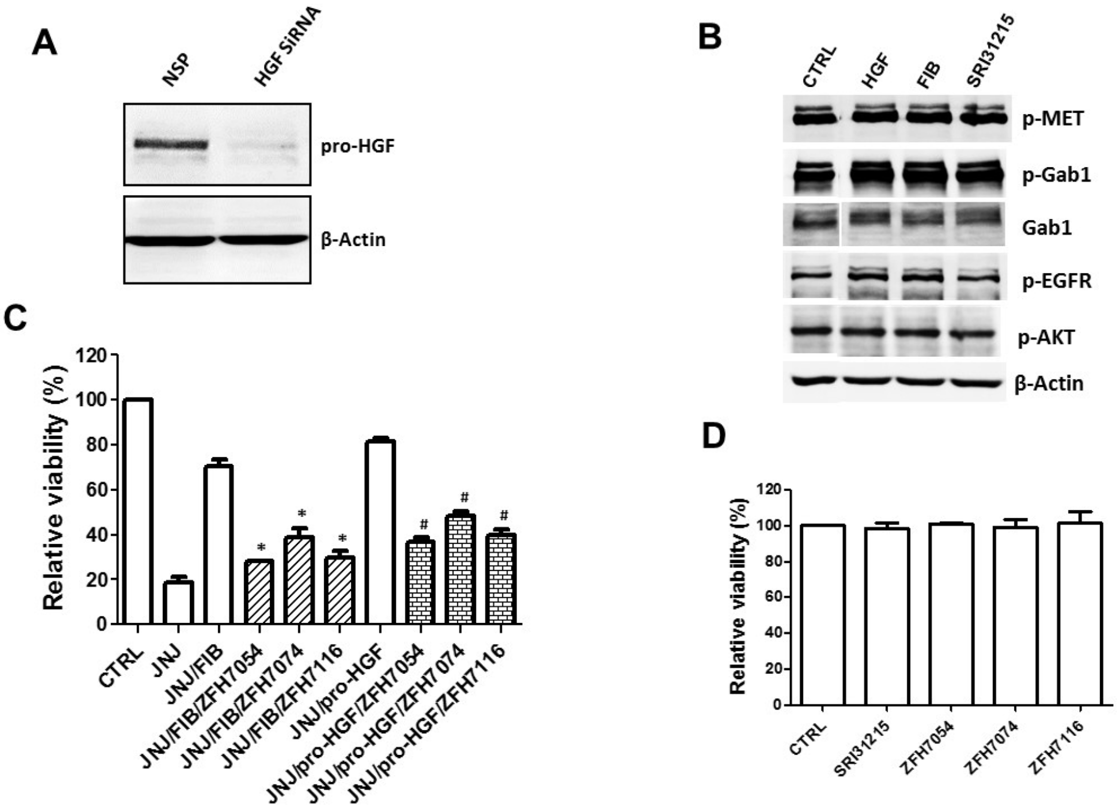

Supplementary Figure 3: Silencing of HGF in WI38 fibroblasts. **(A)** Cell lysates from WI38 fibroblasts transfected with either scrambled, non-specific siRNA (NSP), or HGF-specific siRNA (HGF siRNA) were immunoblotted for HGF. **(B)** The effect of HGF and fibroblasts on MET activation in EBC-1 cells. **(C)** EBC-1 cells were treated with JNJ38877605 (25 nM), conditioned medium from WI38 fibroblasts (FIB), pro-HGF and peptide inhibitors of pro-HGF activation, ZFH7054 (100 nM), ZFH7074 (100 nM) or ZFH7116 (100 nM) as indicated. Cell viability was determined by CellTiter Glo® after 72 h. **(D)** EBC-1 cells were treated with SRI31215 (10 μM), ZFH7054 (100 nM), ZFH7074 (100 nM) or ZFH7116 (100 nM) alone. Cell viability was determined by CellTiter Glo® after 72 h. *,$p < 0.05$ compared to JNJ/FIB; #, $p < 0.05$ compared to JNJ/pro-HGF.

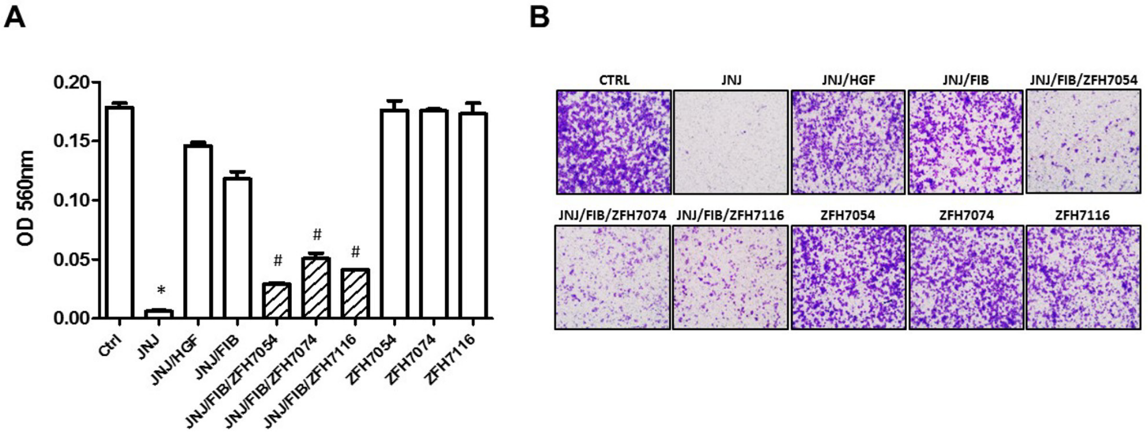

Supplementary Figure 4: Migration of EBC-1 cells was assessed by transwell migration assay after treatment with JNJ38877605 (25 nM) in the absence or presence of recombinant HGF or conditioned medium from fibroblasts (FIB) and ZFH7054 (100 nM), ZFH7074 (100 nM) or ZFH7116 (100 nM) for 24h as indicated. **(A)** Relative migration of cells was determined by the optical density (OD) of stained cells at 560 nm. *, $p < 0.05$ compared to CTRL; #, $p < 0.05$ compared to JNJ/FIB. **(B)** Representative images of stained cells that migrated across the transwell polycarbonate membrane.

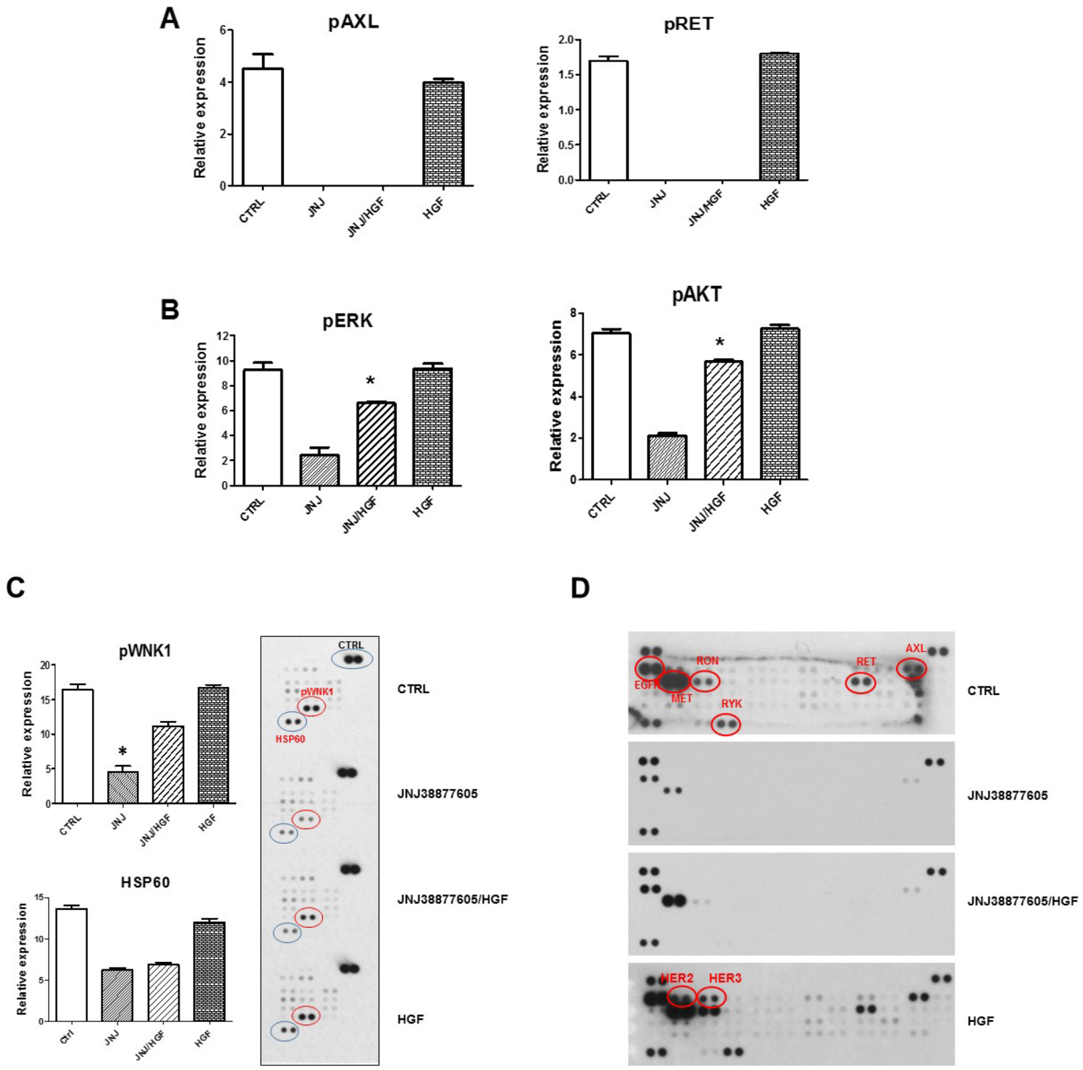

Supplementary Figure 5: HGF signaling in MET- inhibited EBC-1 cells. EBC-1 cells were incubated with JNJ38877605 (25 nM) or recombinant HGF (100 nM) alone, or with JNJ38877605 and recombinant HGF for 6 hours. Cell lysates were prepared and subjected to antibody-based human phospho-receptor tyrosine kinase (RTK) array **(A)** or Phosphokinase array **(B)** to analyze the phosphorylation profiles of receptor tyrosine kinases and their targets. **(C)** Antibody array demonstrating that MET signaling regulates WNK1 activity. Circled are WNK1, HSP60 and positive control (CTRL). *, $p < 0.05$ compared to JNJ3887605 (JNJ). **(D)** Proteome profiler Phospho-Tyrosine Kinase Array of EBC-1 cells treated with JNJ38877605 in the absence or the presence of HGF.
